# Risk of Complications and Survival of Patients Dialyzed with Permanent Catheters

**DOI:** 10.3390/medicina56010002

**Published:** 2019-12-19

**Authors:** Anna Szarnecka-Sojda, Wojciech Jacheć, Maciej Polewczyk, Agnieszka Łętek, Jarosław Miszczuk, Anna Polewczyk

**Affiliations:** 1 Vascular Surgery Clinic, Provincial Hospital, 25-736 Kielce, Poland; j.miszczuk@op.pl; 2 2nd Department of Cardiology, School of Medicine with the Division of Dentistry in Zabrze, Medical University of Silesia in Katowice, 40-055 Katowice, Poland; wjachec@interia.pl; 3 Faculty of Medicine and Health Studies, Jan Kochanowski University, 25-369 Kielce, Poland; Maciek.polewczyk@gmail.com (M.P.); agaletek@gmail.com (A.Ł.); annapolewczyk@wp.pl (A.P.); 4 Acute Cardiac Care Unit, Swietokrzyskie Cardiology Center, 25-736 Kielce, Poland; 5 Department of Cardiology, Swietokrzyskie Cardiology Center, 25-736 Kielce, Poland

**Keywords:** permanent catheter, dialysis fistula, older age, risk of death, complications, survival

## Abstract

*Background and Objectives*: An increase in the incidence of end-stage renal disease (ESRD) is associated with the need for a wider use of vascular access. Although arteriovenous (A-V) fistula is a preferred form of vascular access, for various reasons, permanent catheters are implanted in many patients. *Materials and Methods:* A retrospective analysis of clinical data was carried out in 398 patients (204 women) who in 2010–2016 were subjected to permanent dialysis catheters implantation as first vascular access or following A-V fistula dysfunction. The factors influencing the risk of complications related to vascular access and mortality were evaluated and the comparison of the group of patients with permanent catheter implantation after A-V fistula dysfunction with patients with first-time catheter implantation was carried out. *Results:* The population of 398 people with ESRD with mean age of 68.73 ± 13.26 years had a total of 495 permanent catheters implanted. In 129 (32.6%) patients, catheters were implanted after dysfunction of a previously formed dialysis fistula. An upward trend was recorded in the number of permanent catheters implanted in relation to A-V fistulas. Ninety-two infectious complications (23.1%) occurred in the study population in 65 patients (16.3%). Multivariate analysis showed that permanent catheters were more often used as the first vascular access option in elderly patients and cancer patients. Mortality in the mean 1.38 ± 1.17 years (min 0.0, max 6.70 years) follow-up period amounted to 50%. Older age and atherosclerosis were the main risk factors for mortality. Patients with dialysis fistula formed before the catheter implantation had a longer lifetime compared to the group in which the catheter was the first access. *Conclusion:* The use of permanent catheters for dialysis therapy is associated with a relatively high incidence of complications and low long-term survival. The main factors determining long-term survival were age and atherosclerosis. Better prognosis was demonstrated in patients after the use of A-V fistula as the first vascular access option.

## 1. Introduction

End-stage renal disease (ESRD) is an important medical and social problem, leading to an increase in the number of patients requiring dialysis and kidney transplantation. The concept of a global epidemic of chronic kidney disease (CKD) has appeared, and its incidence is increased mainly in developing countries [1]. About 75% of patients with ESRD require renal replacement therapy and access to dialysis [2,3]. In Poland, approximately 4.5 million people suffer from chronic kidney disease and 21,043 patients receive renal replacement therapy [4]. According to the Kidney Disease Outcomes Quality Initiative (KDOQI), the recommended form of vascular access is arteriovenous fistula (A-V) from the patient’s own vessels and 80%–90% of patients should be dialyzed using it [2,4,5,6,7,8]. Aging of the population and high morbidity in the CKD patients often makes the creation of the A-V fistula impossible and the permanent catheter becomes the only salvage for these patients [3,4]. Dialysis catheters are an alternative to the continuation of renal replacement therapy in patients with existing vascular access failure, when the surgical possibilities for the formation of an A-V fistula have been exhausted. They are also used as bridging therapy for those who are waiting for a live donor transplant and for the maturation of the dialysis fistula formed. Dialysis catheters are also used in people with severe heart failure and respiratory failure, in whom fistula formation would be associated with exacerbation of the underlying disease [9,10,11,12,13,14]. Although the dialysis fistula is the gold standard, in various clinical situations, a catheter inserted into the venous system is selected for dialysis [7,15]. The advantage of catheters is related to obtaining a fast access and the possibility of immediate dialysis and the limitation of high risk of infectious, thrombotic complications, dysfunctions leading to impairment of dialysis, associated with central vascular stenosis. These complications are likely to affect mortality in a catheter-dialyzed patient. Current reports more and more often show that the survival rate of dialyzed patients depends to a large extent on the type of vascular access used. The aim of the present study was to analyze trends in implantation of permanent catheters, compare populations with permanent catheters implanted as the first and subsequent access, and evaluate the risk of complications and mortality of patients dialyzed using permanent catheters during the observation period.

## 2. Materials and Methods

The study included 398 adults whom in the period from the 1 January 2010 to the 31 December 2016 had permanent dialysis catheters implanted for the first time. It was a retrospective study based on the data obtained from the regional branch of the National Health Fund and hospital medical documentation. The study group included patients who had a tunneled catheter inserted as means of first-time access and patients with a catheter implanted due to dysfunction of a previously developed dialysis fistula. The performed procedures of dialysis access and the diseases that led to the permanent catheter insertion were evaluated for the whole study group. The International Statistical Classification of Diseases and Related Health Problems (ICD10) unit codes were used for the analysis. The influence of the cause of implantation of the dialysis catheter on the time of its functioning was assessed. Based on this data, the frequency of vascular access implantation was assessed over the observation period. The catheter functioning time was compared in two groups: the first one was comprised of catheters inserted as a result of the initial renal reasons (these were diseases coded according to ICD 10 with codes: N03.9; N04.0; N10; N12.0; N13.2; N17.0; N17.8 N17.9; N27.0), the second consisted of those that were inserted as a result of initial nonrenal reasons (these are diseases according to ICD10: A41.0; A41.1; A41.5; E11.2; E11.5; I70.2; I70.9; I71.4; S68.8; T82.3). The analysis included clinical and procedural factors potentially affecting the risk of developing vascular access complications and mortality. The comparison of the group of patients with permanent catheter implantation after dysfunction of the dialysis fistula with patients with first-time catheter implantation was carried out. The procedural factors analysis included: reasons for permanent catheter insertion and removal; reasons for further hospitalizations once the catheter was implanted; impact of the disease that led to catheter insertion on its functioning time; the number of catheters implanted in one patient; time of functioning of the first catheter; functioning time of a catheter implemented after previously developed dialysis fistula. The catheter functioning time was calculated from the moment of its implantation until its removal, replacement, patient’s death or the end of the study. Survival time was calculated from the beginning of the study to its end or the patient’s death.

### 2.1. Statistical Methods

The distribution of continuous variables was assessed using the Shapiro–Wilk test. Values of continuous variables are presented as mean ± SD and number (%) for dichotomous variables.

Student’s *t*-test was used to compare continuous variables of parametric distribution. Variables with nonparametric distribution were compared with the Mann–Whitney "U" test, while the dichotomous variables-with the Chi^2^ test with the Yates correction. The Kruskal–Wallis ANOVA test was used to analyze the influence of the disease classification determining the need for renal replacement therapy for the duration of the catheter’s life.

To determine the risk factors for death during the follow-up period, the uni- and multifactorial Cox regression model was used. The analysis of factors influencing the selection of dialysis access options (catheter or fistula) was performed using uni- and multifactorial logistic regression. Multivariable analyses included variables whose differences in parametric and nonparametric tests reached *p* < 0.05.

Additionally, the Kaplan–Meier survival curves were plotted for groups of patients determined on the basis of the first-time dialysis access type. Differences in the course of the curves were assessed by the log rank test. Statistical significance was determined for *p* < 0.05.

### 2.2. Ethical Statement

The study was carried out in accordance with the Declaration of Helsinki and was approved by the Bioethics Commission of Regional Medical Board (Świętokrzyska Izba Lekarska, permit no. 21/2017; approved on 27 April 2017).

## 3. Results

**Figure 1:**
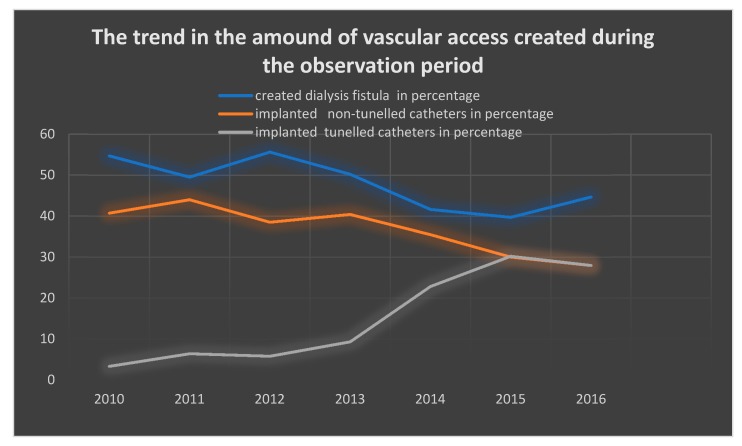
Percentage of tunneled, nontunneled catheters, and dialysis fistulas during the observation period.

The study included 398 adults (204 women, 51.25%) of mean age 68.73 ± 13.26 years, who had a permanent catheter implanted for the first time. In total, 495 catheter implantations had been registered during the mean 1.38 ± 1.17 years (min 0.0 max 6.70 years) follow-up period. During that time, a significant increase in the frequency of permanent catheters implantation was observed. The percentage of implanted catheters in relation to developed dialysis fistulas increased from 3.35% in 2010 to 27.98% in 2016. Detailed analysis confirmed a linear upward trend in the number of implanted tunneled catheters, as seen in Figure 1.

In the studied population, in 129 patients (32.16%), permanent catheters were implanted after A-V fistula dysfunction. In 322 (80.9%) patients, only a single catheter was implanted during the observation period, and 58 (14.57%) patients required at least one catheter replacement. The need to replace the catheter was more frequent in women (*p* < 0.05), as seen in Table 1.

The etiology of renal failure did not affect the functioning time (age) of the first catheter, the functioning time of all catheters in one patient and the mean functioning time of one catheter (according to Kruskal–Wallis ANOVA *p* = 0.181 for the first catheter age, *p* = 0.485 for the age of all catheters, and *p* = 0.267 for the mean age of the catheter).

The reason for catheter implantation (due to initial renal disease or other nonrenal reasons) had no effect on the duration of its functioning time (according to Kruskal–Wallis ANOVA *p* = 0.3015 for the age of the first catheter, *p* = 0.535 for the age of all catheters, *p* = 0.148 for the mean age of the catheter).

The most common diseases associated with renal failure in the study group were hypertension (27.4%), generalized atherosclerosis (27.1%), heart failure (19.6%), and cancer (19.6%). There were no differences in the incidence of comorbidities between men and women. Ninety-two infectious complications (23.1%) in 65 patients (16.3%) were reported (mean infection ratio was 0.46 per 1000 catheter days) in the study population with the lower frequency of infection in the female population (*p* < 0.05), as seen in Table 1.

Patients with initially formed A-V fistula were younger, with less frequent diabetes mellitus and generalized atherosclerosis. The incidence of cardiovascular disease and number of infectious complications related to catheters was comparable to patients with a permanent catheter implanted as a first access. The total duration of functioning of all catheters was 182,613 days; the mean duration of one catheter functioning was 436 ± 398. The duration of functioning of catheters implanted after dysfunction of the dialysis fistula was longer compared to those implanted as the first-time access (565 days ± 409 vs. 476 days ± 434; *p* = 0.01), although the number of thrombotic complications and the need to replace catheters was more frequent in this group of patients. In patients with a permanent catheter as the first vascular access, diabetes, generalized atherosclerosis, and neoplastic disease were more common. In this group a tendency to higher mortality was also more frequent, as seen in Table 2.

Univariate logistic regression showed that the choice of a permanent catheter as the first option for dialysis access is determined by older age of the patient, diseases of the cardiovascular system of atherosclerotic etiology, and neoplastic disease (borderline statistical significance). Multivariate analysis confirmed the predictive effect of the age of patients and neoplastic disease (borderline statistical significance), as seen in Table 3.

Analysis of the survival rate of the entire study population of dialysis patients using permanent catheters showed that during the mean 1.38 ± 1.17 years follow-up period, 199 (50%) people died: 103 (50.49%) women and 96 (49.48%) men. Patients who died were older, with frequent generalized atherosclerosis. Catheter replacement had no effect on mortality; however, the time from the first implantation and the mean time of catheter functioning was longer in patients who survived the observation period, as seen in Table 4.

Cox univariate regression analysis demonstrated that death risk factors for dialysis patients using permanent catheters included older age and the presence of generalized atherosclerosis. In the two-factor assessment, only the influence of older age was confirmed, as seen in Table 5.

**Figure 2:**
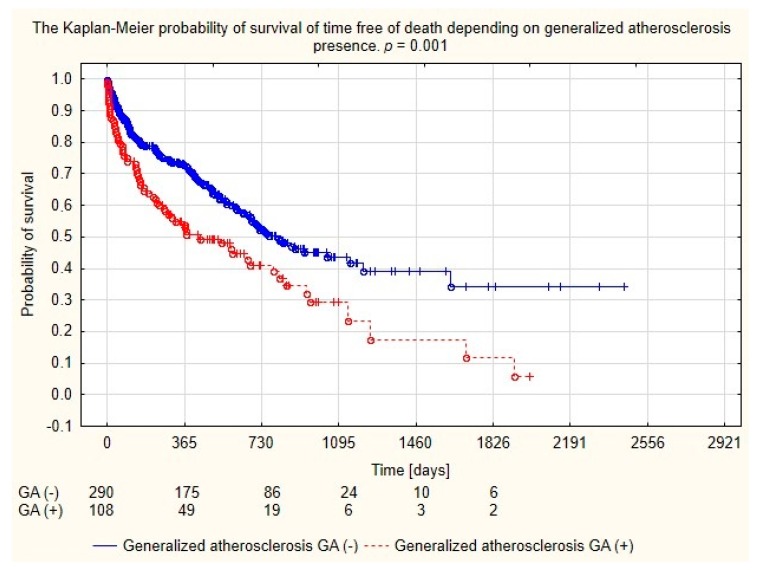
Probability of survival depending on the generalized atherosclerosis in the population of patients dialyzed with permanent catheters.

Analysis of the long-term survival of dialyzed patients demonstrated higher mortality of patients with generalized atherosclerosis, as seen in Figure 2.

**Figure 3:**
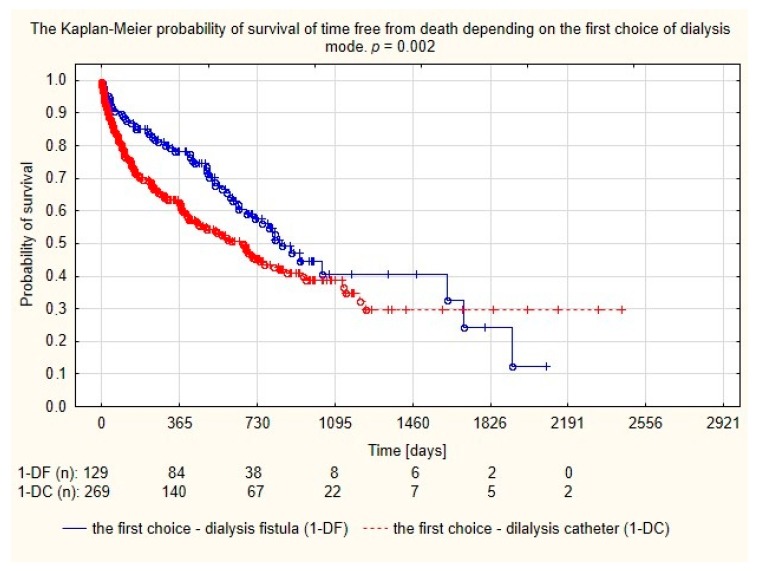
Probability of survival depending on the first choice of dialysis mode.

Assessment of survival probability using Kaplan–Meier curves showed that patients with dialysis fistula formed before the catheter implantation had a longer lifetime compared to the group in which the catheter was the first access, as seen in Figure 3.

## 4. Discussion

On the basis of the current analysis of clinical data of patients undergoing dialysis in 2010–2016, it was proved that despite the KDOQI’s recommendations for limiting the number of implanted catheters in relation to dialysis fistulas, their percentage is still increasing. The study showed that during the observation period, it increased from 3.35% in 2010 to 27.98% in 2016.

The current literature includes few reports on the percentage of permanent catheters in dialysis therapy in Poland and in the world, but all of them confirm the increasing tendencies in the use of this form of vascular access. In one of the studies from south-eastern Poland conducted in 2006–2010, the percentage of patients dialyzed with a long-term catheter increased from 14.35%–20.5% [16]. The upward trend continues, and according to the data from Fresenius Nefrocare Polska presented at the First Congress of Polish Vascular Access to Dialysis Club in 2017, the number of patients dialyzed in Poland increased from 2345 in 2010 to 6112 in 2017. The data also showed an increase in the share of permanent vascular catheters from 17.7% in 2010 to 30.3% in 2017. This does not differ from the data presented in global reports. According to Dialysis Outcomes and Practice Patterns Study (DOPPS) in 2009–2011 in Europe, the percentage of catheters ranged from 12%% to 32%, and in Canada it reached 49% [17]. This tendency is probably associated with an increase in life expectancy and a high rate of morbidity in the group of dialysis patients. They are much more often burdened with cardiological, pulmonary, and oncological complications as well as diabetes, and the arteriovenous fistula often poses an additional risk of deteriorating health through burden of the circulatory system. Moreover, due to changes in the vessel wall in the elderly as a result of diabetes, atherosclerosis, and calcification, the creation of preferred access, i.e., A-V fistula, is often impossible, which is why permanent catheters were implanted more often in older patients with these comorbidities [18,19,20,21,22]. The catheter functioning time in the current study was similar to the period presented in the available publications. One of the papers compares the operating time of tunneled catheters installed first to those installed after the previous nontunneled catheters. The mean functioning duration of catheters in this material was 370 days, with a median of 287 days, and was comparable in both groups [16,23,24]. Another work showed a significant advantage on the functioning time of catheters implanted as first-time over the times of those implanted second [25]. The functioning duration of the catheter also depended on its location; e.g., in the right internal jugular vein, it was longer than in other locations. Nondiabetic patients had longer catheter operation times in relation to diabetic patients, and the time of catheter functioning was affected by the type of material used for their construction. The age and sex of the patient as well as the operator’s skills did not influence the catheter operation times [16,25,26]. In the present study, sex, location of the catheter, and the reasons for the catheter installation had no effect on its functioning time. This time was longer in the group of patients in whom the catheter was installed after the previous fistula as the next access in comparison with the catheter installed as the first access (565.248 vs. 476.342 days; *p* < 0.001). Patients in the group with an earlier fistula were younger and atherosclerosis, diabetes and neoplastic diseases were less common, which in comparison to the second group could have influenced the longer maintenance of the functioning of the catheter. All these factors contributed to the improved survival of patients in whom A-V fistula was used as the first vascular access.

The presented study identified 92 (23.1%) catheter-related infections of an average infection rate of 0.46 per 1000 catheter days. In the case of tunnelled catheters, according to various sources, frequency of infection ranges from 1.6 to 5.5 occurrences per 1000 catheter days [22,27]. Another observation of a large number of patients showed an infection rate of 0.514 per 1000 catheter days [28]. Another study indicated the impact of catheter location on the frequency of catheter-related infections and its dysfunction [29]. The present study showed that in the group of infected catheters, catheter location had no impact on operation time.

Some reports in the literature show a high risk of infection in patients dialyzed by catheter, compared to those using A-V fistulas. In one of them, infectious complications in dialysis patients using permanent catheters amounted to approximately 40% [30,31]. The present study demonstrated no difference in the number of infections between the patients dialyzed with catheters after dysfunction of the previously created fistula and those dialyzed first with a catheter. In the presented study, 50% of mortality was observed during the mean 1.38 ± 1.17 years follow-up period. These data are comparable to other reports. According to various sources, the survival rate in the dialysis group is 67% after two years of dialysis, 35% after five years, and 11% after 10 years [19]. Mortality in the dialysis patients depends to a large extent on the type of vascular access used. It has been proven that dialysis fistulas made of vascular prostheses and central catheters increase the risk of death [7,31,32,33,34,35]. One of the current publications showed two-fold higher mortality in patients dialized by catheter (22.7%) compared to the fistula group (12.2%), and increased hospitalization rates in the catheter group. Higher mortality was observed in the group of dialyzed older patients, over 65 years of age, and in patients with diabetes. Patients dialyzed with catheters had a 50% higher risk of death than those dialyzed with fistula [36]. The main causes of death in this study were cardiac complications, occurring in 9.4%, whereas infections occurred only in 2.9% of subjects. Another study of the 2666 patients dialyzed between 2009 and 2011 showed that 873 (32%) patients died during the follow-up period. Patients using tunneled catheters demonstrated, in relation to other forms of access, a higher risk of death from all causes of exposure to renal replacement therapy (HR = 1.83–2.08). Higher risk of death from cardiovascular causes (corrected HR: 2.20–2.95) and death from infection (corrected HR: 3.10–3.336) were also reported [14]. In general, cardiovascular disease caused by atherosclerotic lesions in the vessels was considered to be the main cause of death in patients undergoing dialysis in the course of ESRD [37,38]. In our study, atherosclerosis was the main risk factor of mortality, but the survival was mainly determined by the age of the patient.

## 5. Study Limitations

The current study was based on retrospective data of medical procedures, which limits the full clinical interpretation. It would be advisable to perform a prospective study with greater access to medical records in order to search for new risk factors of complications and mortality in this very burdened group of dialysis patients.

## 6. Conclusions

The use of permanent catheters for dialysis therapy is associated with low long-term survival rate, especially in patients in whom this catheter was used as the first vascular access option. Better prognosis of patients with A-V fistula at baseline has been documented by demonstrating a longer dialysis duration. The main factor determining long-term survival was age, and the significant impact of atherosclerosis on mortality was confirmed.

## Figures and Tables

**Table 1:** Clinical characteristics of the studied population.

	All Patients	Women	Men	*p*-Value
Number of patients (*n,* %)	398 (100%)	204 (51.25%)	194 (48.75%)	NS
Age (years, SD)	68.73 ± 13.26	69.50 ± 13.22	67.89 ± 13.28	NS
The number of catheters inserted after dysfunction of previously formed dialysis shunt (*n*, %)	129 (32.41)	70 (34.31)	59 (30.31)	NS
The number of patients with one catheter (*n*, %)	322 (80.90%)	160 (78.43%)	162 (83.51%)	NS
The number of patients requiring one catheter replacement (*n*, %)	58 (14.57%)	37 (18.14%)	21 (10.82%)	<0.1
The number of patients requiring two or more catheter replacements (*n*, %)	18 (4.52%)	7 (3.33%)	11 (5.67%)	NS
Time from first implantation of the catheter to the end of the study (days, SD)	505.2 ± 428.3	508.9 ± 424.8	501.18 ± 433.0	NS
The mean time of catheter functioning (days, SD)	435.7 ± 398.2	439.7 ± 404.6	431.54 ± 392.4	NS
The number of catheters replaced due to infection (*n*, %)	10 (2.51)	6 (2.94)	4 (2.06)	NS
Generalized atherosclerosis (*n*, %) ^1^	108 (27.14%)	51 (25.00%)	57 (23.98%)	NS
Thrombotic catheter complications (*n*, %)	66 (16.58%)	38 (18.63%)	28 (14.43%)	NS
Diabetes (*n*, %)	73 (18.34%)	32 (15.69%)	41 (21.13%)	NS
Coronary artery disease (*n*, %)	44 (11.06%)	23 (11.27%)	21 (10.82%)	NS
Hypertension (*n*, %)	109 (27.39%)	53 (25.98%)	56 (28.87%)	NS
Heart failure III-IV acc. to NYHA (*n*, %)	78 (19.60%)	35 (17.16%)	43 (22.16%)	NS
Stroke in the history (*n*, %)	4 (1.01%)	3 (1.47%)	1 (0.52%)	NS
Chronic atrial fibrillation (*n*, %)	12 (3.02%)	7 (3.43%)	5 (2.58%)	NS
Neoplastic disease (*n*, %)	78 (19.60%)	35 (17.16%)	43 (22.16%)	NS
Other diseases (*n*, %) ^2^	87 (21.86%)	41 (20.60%)	46 (23.12%)	NS
The number of all local and generalized infectious complications (*n*, %)	92 (23.12%)	36 (17.65%)	56 (28.87%)	*p* < 0.05
The number of patients with local and generalized infectious complications (*n*, %)	65 (16.33%)	29 (14.22%)	36 (18.56%)	NS
Decease (*n*, %)	199 (50.00%)	103 (50.49%)	96 (49.48%)	NS

^1^ Atherosclerosis diagnosed at various locations: cerebral arteries, aorta, and visceral and renal arteries, arteries of the lower and upper extremities described in in the medical documentation with codes according to ICD10 classification: I70.0, I70.1, I70.2, I70.8, I70.9, I65.2. ^2^ diseases occurring in patients in the study group not separately analyzed, recorded with codes according to ICD10 classification: D 50, D53, D64, E03, E04 E27, G65, J20, J42, J44, J45, J 81, J 96, K26, K50, K65, K76, L08, L97, M06, M 32, M 34, S68.

**Table 2:** Comparative analysis of clinical and procedural parameters of patients with permanent catheters implanted after A-V fistula and implanted as the first access.

	Patients with a Permanent Catheter Implanted after Dysfunction of a Previously Created Dialysis Fistula	Patients with a Permanent Catheter Implanted as the First Access	*p*-Value
Number of patients (*n*, %)	129 (32.41)	269 (67.59)	
Age (years, SD)	66.202 ± 11.799	69.944 ± 13.765	<0.001
Female (*n*, %)	70 (54.26)	134 (49.81)	NS
Time from the first catheter implantation to the end of the follow-up (days, SD)	565.248 ± 409.284	476.342 ± 434.248	<0.001
Mean time of catheter functioning (days, SD)	449.721 ± 353.274	429.037 ± 418.505	NS
The number of patients with one catheter (*n*, %)	92 (71.32)	230 (85.50)	0.001
The number of patients requiring one catheter replacement (*n*, %)	26 (20.16)	32 (11.90)	0.042
The number of patients requiring two or more catheter replacements (*n*, %)	11 (8.53)	7 (2.60)	0,016
Thrombotic catheter complications (*n,* %)	30 (23.26)	36 (13.38)	0.020
Diabetes (*n*, %)	19 (14.73)	54 (20.07)	0.001
Generalized atherosclerosis (*n*, %)	26 (20,16)	82 (30,48)	0,041
Coronary artery disease (*n*, %)	12 (9.30)	32 (11.90)	NS
Hypertension (*n*, %)	30 (23.26)	79 (29.37)	NS
Heart failure III-IV acc. to NYHA (*n*, %)	27 (20.93)	51 (18.96)	NS
Stroke in the history (*n*, %)	2 (1.55)	2 (0.74)	NS
Atrial fibrillation (*n*, %)	3 (2.33)	9 (3.35)	NS
Neoplastic disease (*n*, %)	17 (13.18)	61 (22.68)	0.036
The number of all local and generalized infectious complications (*n*, %)	34 (26.36)	58 (21.56)	NS
The number of patients with local and generalized infectious complications (*n*, %)	26 (20.16)	39 (14.49)	NS
Decease (*n*, %)	56 (43.41)	143 (53.16)	NS (0.087)

**Table 3:** Univariate and multivariate logistic regression of the probability of the first-time permanent catheter implantation.

**Univariate Logistic Regression**			
**Parameter**	**HR**	**95% CI**	***p*** **-Value**
Patient’s age	1.021	1.005–1038	0.009
A disease of atherosclerotic etiology (yes/no) ^1^	1.737	1.049–2.875	0.031
Diabetes	1.454	0.820–2.578	NS
Neoplastic disease	1.932	1.075–3.473	0.027
Other diseases ^2^	1.672	0.969–2.885	NS (0.064)
Heart failure III-IV according to NYHA classification	0.884	0.523–1.493	NS
**Multivariate Logistic Regression**			
Patient’s age	1.017	0.999–1.036	NS (0.060)
A disease of atherosclerotic etiology ^1^ (yes/no)	1.278	0.722–2.263	NS
Neoplastic disease	1.728	0.950–3.143	NS (0.073)
Other diseases ^2^	1.531	0.868–2.700	NS

^1^ Atherosclerosis diagnosed at various locations: cerebral arteries, aorta, and visceral and renal arteries, arteries of the lower and upper extremities described in in the medical documentation with codes according to ICD10 classification: I70.0, I70.1, I70.2, I70.8, I70.9, I65.2. ^2^ diseases occurring in patients in the study group not separately analyzed, recorded with codes according to ICD10 classification: D 50, D53, D64, E03, E04 E27, G65, J20, J42, J44, J45, J 81, J 96, K26, K50, K65, K76, L08, L97, M06, M 32, M 34, S68.

**Table 4:** Comparative analysis of clinical and procedural parameters of patients, including the division into patients alive and deceased during the observation period.

	Study Group	Decease	Living Patients	*p*-Value
The number of patients (*n*)	398	199	199	
Sex (women) (*n*, %)	204 (51.26)	103 (51.76)	101 (50.75)	NS
Patient’s age (years ± SD)	68.88 ± 13.29	72.12 ± 11.65	67.18 ± 13.64	<0.001
Presence of an earlier dialysis fistula (*n*, %)	129 (32.41%)	56 (28.15%)	73 (36.68%)	NS (0.086)
The number of patients with one catheter (*n,* %)	322 (80.90%)	163 (81.91%)	159 (79.90%)	NS
The number of patients requiring one catheter replacement (n, %)	58 (14.57%)	27 (13.57%)	31 (15.58%)	NS
The number of patients with two or more catheter replacements (*n,* %)	18 (4.52%)	9 (4.52%)	9 (4.52%)	NS
The number of catheters replaced due to infection (*n*, %)	10 (2.51)	5 (2.51%)	5 (2.51%)	NS
The number of catheters replaced due to dysfunction (*n*, %)	66 (16.58)	33 (16.58%)	33 (16.58%)	NS
Time from the first catheter implantation to the end of the study (days, SD)	505.2 ± 428.3	317.0 ± 342.7	693.3 ± 423.2	<0.001
Mean time of catheter functioning (days, SD)	435.7 ± 398.2	256.6 ± 270.8	614.9 ± 424.4	<0.001
Thrombotic catheter complications (*n*, %)	66 (16.58%)	33 (16.58%)	33 (16.58%)	NS
Infectious complications (*n*,%)	65 (16.33%)	35 (17.59%)	30 (15.08%)	NS
Diabetes mellitus (*n*, %)	58 (14.57%)	28 (14.07%)	30 (15.08%)	NS
Generalized atherosclerosis (*n*, %)	108 (27.14%)	67 (33.67%)	41 (20.60%)	<0.01
Coronary artery disease (*n*, %)	44 (11.06%)	22 (11.06%)	22 (11.06%)	NS
Hypertension (*n*, %)	109 (27.39%)	56 (28.14%)	53 (26.63%)	NS
Atrial fibrillation (*n*, %)	12 (3.02%)	6 (3.02%)	6 (3.02%)	NS
Heart failure II/IV according to NYHA classification (*n*, %)	78 (19.60%)	43 (21.61%)	35 (17.59%)	NS
Stroke in the history (*n*, %)	4 (1.01%)	3 (1.51%)	1 (0.50%)	NS
Neoplastic disease (*n*, %)	78 (19.60%)	36 (18.09%)	42 (21.11%)	NS

**Table 5:** Risk factors of death. Cox uni- and multivariate logistic regression.

**Decease Risk Cox-Univariate**	**HR**	**95% CI**	***p*** **-Value**
Patient’s age	1.035	1.023–1.049	0.000
Generalized atherosclerosis	1.662	1.238–2.232	0.000
**Decease Risk Cox-Bivariate**	**HR**	**95% CI**	***p*-Value**
Patient’s age	1.034	1.020–1.050	0.000
Generalized atherosclerosis	1.062	0.752–1.500	NS
